# Dynamics of hospital admissions and all-cause mortality of HIV infected patients in Kazakhstan: data from unified nationwide electronic healthcare system 2014–2019

**DOI:** 10.3389/fpubh.2023.1138604

**Published:** 2023-06-20

**Authors:** Kamilla Mussina, Anara Abbay, Yesbolat Sakko, Dmitriy Syssoyev, Arnur Gusmanov, Ainur Abdrakhmanova, Aiymkul Ashimkhanova, Abduzhappar Gaipov

**Affiliations:** ^1^Department of Medicine, School of Medicine, Nazarbayev University, Astana, Kazakhstan; ^2^Clinical Academic Department of Internal Medicine, CF “University Medical Center”, Astana, Kazakhstan

**Keywords:** HIV, mortality, incidence, prevalence, survival

## Abstract

**Background and objectives:**

Although global HIV pandemic has stabilized, it continues to rise in Eastern Europe and Central Asia due to exponential growth of newly acquired cases. Based on UNAIDS, there are currently 35,000 people living with HIV (PLWH) in Kazakhstan. This alarming HIV epidemiologic situation mandates urgent investigation of causes, routes of transmission and other characteristics in order to halt the epidemic. We aimed to analyze the data of all hospitalized patients for the period of 2014–2019 who tested positive for HIV from the Unified National Electronic Health System (UNEHS) of Kazakhstan.

**Methods:**

This cohort study extracted data for all HIV positive patients during 2014–2019 from UNEHS of Kazakhstan to apply descriptive, Kaplan–Meier estimation, and Cox proportional hazards regression model. Crosscheck of the target population data was conducted with tuberculosis, viral hepatitis, alcohol abuse and intravenous drug user (IDU) cohorts in order to create a comprehensive database. All survival functions and factors associated with mortality were tested for significance.

**Results:**

The cohort population (*n* = 2,213) mean age was 33.3 ± 13.3 years with 1,375 males (62.1%) and 838 females (37.9%). Incidence rate decreased from 2.05 in 2014 to 1.88 in 2019, however, prevalence and mortality continues to escalate every year, the mortality raised significantly from 0.39 in 2014 to 0.97 in 2019. People aged >50 years, males, retired people, patients from tuberculosis hospital profile had much lower survival probabilities than the corresponding groups. Adjusted Cox regression model death hazard showed strong association of HIV patients with tuberculosis coinfection (HR 1.4, 95% CI 1.1; 1.7, *p* < 0.001).

**Conclusion:**

The results of this study demonstrate high rates of HIV mortality, strong association of HIV with TB coinfection, regional, age specific, gender, hospital profile and social status differences that significantly affect HIV prevalence. Since the prevalence of HIV is continuing to increase, more information is necessary for evaluation and implementation of prevention procedures.

## 1. Introduction

Human immunodeficiency virus disease is a retrovirus that affects CD4 counts and leads to acquired immunodeficiency and death. Since its first discovery in 1983, HIV rapidly became an epidemic affecting every country in the world, with the most disastrous impact in Subarian Africa, accounting for half of the global cases. Based on the UNAIDS report, 38 million people live with HIV, and 680 thousand lives were lost due to AIDS-related illnesses. Although the global situation of HIV is stabilized and new cases decreasing for the past decade, in Eastern Europe and Central Asia the epidemic accelerating its growth with newly acquired HIV increased to 72% (1).

Kazakhstan is a developing multi-ethnic country that is largely situated in Central Asia, and a small part in Eastern Europe. It was part of the Soviet Union until 1991. First cases of HIV/AIDS in Kazakhstan are unknown, but probably emerged in the early 1990s after the collapse of the Soviet Union and the country gaining its independence. Open international borders and unstable economic situation led to cross border labor migration, which promoted spread of infection. The primary route of transmission of HIV in Kazakhstan is intravenous drug use as in most Central Asia countries, attributed to geographical region en-route of drug-trafficking from Afghanistan, domestic cultivation of opiates in the setting of unfavorable socioeconomic status (2). Followed by high-risk behavior such as sex-workers, low condum use and STDs. Other routes are less common, but transfusion-related nosocomial outbreak of HIV in children is one the notable HIV outbreaks in 2006 that made a major historical turn in HIV health policy of the country (3).

HIV prevalence in Kazakhstan significantly differs depending on the source of data obtained. Based on UNAIDS, there are currently 35,000 people living with HIV (PLWH) in Kazakhstan, with prevalence of 0,3%. There were 3,600 newly acquired cases in 2020, which is a 73% increase for the past decade indicating exponential increase of epidemic in the country (4). While the local sources such as Kazakh Scientific Center of Dermatology and Infectious diseases reports 26,709 PLWH, 3342 people newly acquired HIV in 2020, with similar prevalence of 0,3% (5).

Despite the alarming numbers of HIV epidemic in Kazakhstan, published data on analysis of clinical presentation, characteristics of HIV, adherence to ART and outcomes of HIV in Kazakhstan is scarce. Analysis of specific sub-population such as HIV and Tuberculosis patients in Almaty region (6), and sociodemographic studies on intravenous drug-uses had been published (7). But overall, it remains a little investigated subject and a significant research gap prevails in understanding the long-term outcome of the people living with HIV.

With a goal of filling that gap, we investigated all hospitalized patients for the period of 2014–2019 who tested positive for HIV. The data was drawn from Unified National Electronic Health System (UNEHS), which provides comprehensive data from major hospitals from all regions of Kazakhstan. To our knowledge, this is the first study describing detailed sociodemographic characteristics of HIV, coinfection with Tuberculosis and viral hepatitis and all-cause mortality of HIV patients who are admitted to the hospital in Kazakhstan.

## 2. Methods

### 2.1. Study design and settings

In this retrospective study, data on HIV-infected people who were admitted to the hospital from 2014–2019 were obtained from UNEHS of Kazakhstan. These are the hospital admissions of HIV-infected patients due to different complications or comorbidities. Data included demographic characteristics, clinical (hospitalization period, stay outcome, treatment outcome, hospital profile. Hospital profiles were classified as infectious, internal medicine and others, which included pediatric, therapeutic and other profiles (77.3%); whereas tuberculosis profiles were classified separately (22.3%)) and behavioral factors (social status) along with the crosscheck of the target population data with tuberculosis, viral hepatitis, alcohol abuse and intravenous drug user (IDU) cohorts in order to create a comprehensive database. Matching the same patients in different databases was conducted by using unique RpnID numbers assigned to each patient registered in a UNEHS registry. Death was defined as outcome variable, and other demographic, clinical and behavioral variables were defined as explanatory variables. For quantitative variables interval scale was used, whereas for qualitative variables (yes/no) categories were used.

### 2.2. Study population

**Figure 1:**
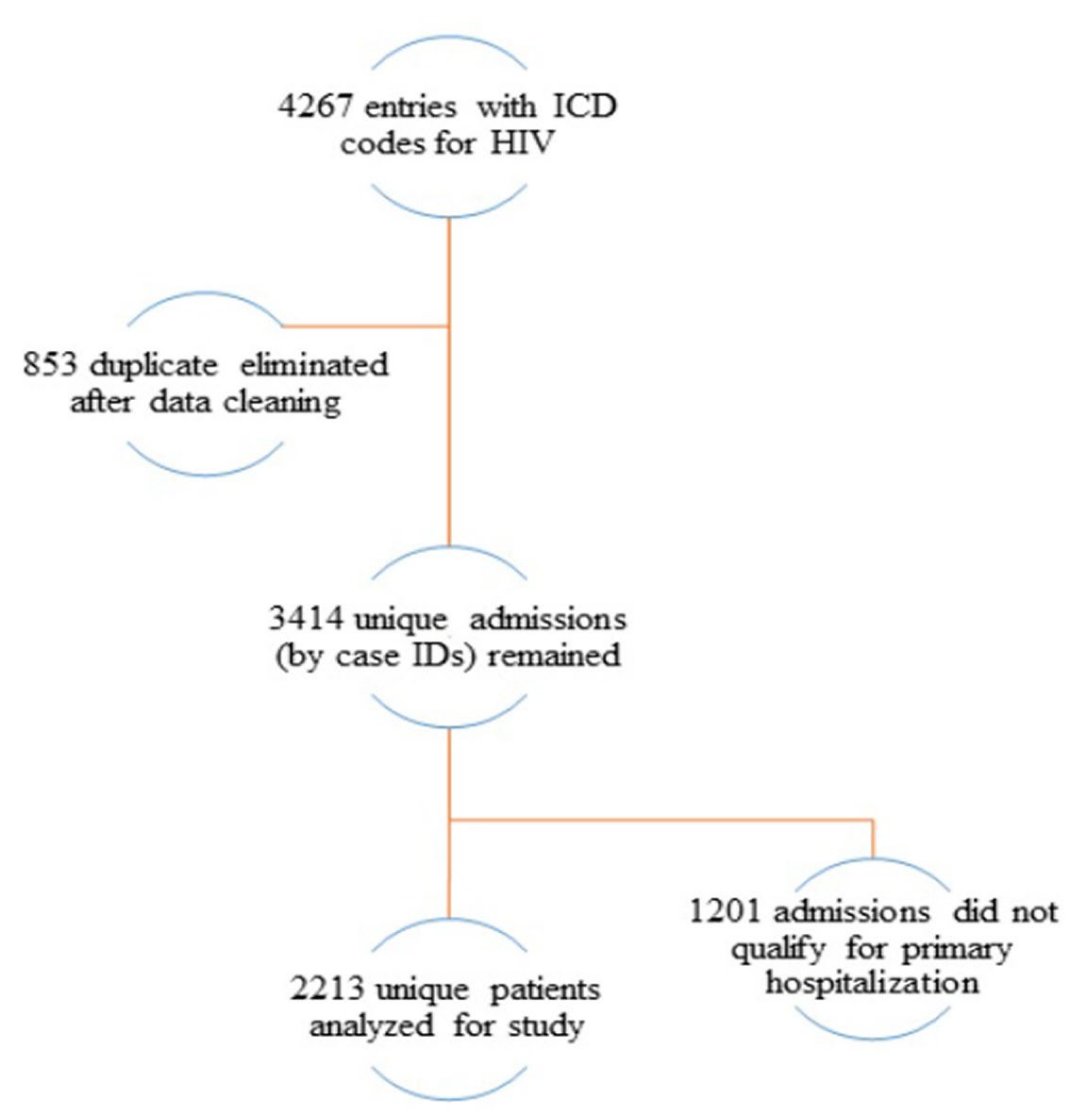
Flow chart diagram of cohort set-up.

Cohort included data of all adult patients and patients less than 18 years of age with HIV infection registered in the electronic registry for the period of 2014–2019. Patients were selected by first recorded HIV infection diagnosis or by first hospital admission and ended at the date of discharge prior December 31, 2019 or, at the date of death. All remaining observations were censored. The data cleaning and management procedures are represented in the flow chart (Figure 1), where only 2,213 unique patients remained with primary hospital admissions.

### 2.3. Statistical analyses

Statistical analyses were conducted using Stata MP2 16.1 version. Descriptive analyses (mean and standard deviation were used for continuous variables, frequencies and relative frequencies were used for categorical variables) were performed to describe the socio-demographic, clinical and behavioral characteristics of the study population. Two sided t-tests for parametric and Mann–Whitney U tests for nonparametric data were performed to compare means. Pearson’s chi-square tests were used for comparison of proportions. For time-to-event analysis Kaplan Meier survival function was used to define survival probabilities of HIV infected patients. For the parameters the date of admission until the date of death was set. Different independent groups were statistically compared by log rank test.

The Cox proportional hazards (PH) regression analysis was performed to estimate unadjusted and adjusted socio-demographic, clinical and behavioral hazard functions for prediction of survival probabilities of HIV-infected patients as well as investigation of associations with other risk factors such as tuberculosis, viral hepatitis, alcohol abuse and intravenous drug use. Magnitude of hazard ratios (HR) and the width of their 95% confidence intervals (CI) were considered in order to decide whether associations are statistically and clinically significant.

Incidence rate, prevalence, mortality rate were calculated for all HIV registered regions of Kazakhstan based on available data from hospital admissions. According to that, maps of Kazakhstan with HIV-infected patients were created for visualization of incidence rate, prevalence and mortality rate in different regions of Kazakhstan using QGIS 3.14 software. In further analysis regions were classified into 5 categories: west, east, south, north and central Kazakhstan. Mangystau, Atyrau, Aktobe belong to West Kazakhstan region; Kostanay, Akmola, Pavlodar and Nur-Sultan city belong to North Kazakhstan; Karaganda belongs to Central Kazakhstan; Kyzylorda, Zhambyl, Almaty along with Almaty city, Shymkent city belong to South Kazakhstan region and East Kazakhstan region.

### 2.4. Ethical approval

The study was approved by the Institutional Review Ethics Committee (NU-IREC 315/21092020 on 23/09/2020) of Nazarbayev University, with exemption from informed consent.

## 3. Results

### 3.1. General characteristics

Table 1 represents the sociodemographic, clinical and behavioral characteristics by gender in the HIV-infected cohort. The cohort consisted of 2,213 patients diagnosed with HIV in their first hospital admissions for the 6 year period (2014–2019). 62% of the cohort (*n* = 1,375) were men. The mean age of HIV-infected patients was 33.3 ± 13.3 years. The largest proportion (almost 38%) of the cohort were middle aged people, aged 35–44 years. 49.3% of HIV-infected patients were Russian (*n* = 1,092) and most of the patients live in urban areas (73.1%). The vast majority of patients (77.3%) were admitted to hospital urgently. All-cause mortality for the cohort was almost 40%. In regards to social status, a little over half of HIV-infected patients were unemployed (almost 55%). Moreover, 479 (21.6%) patients had tuberculosis diagnosis, 368 (16.6%) patients had alcohol abuse and IDU and only 113 (5.1%) patients had viral hepatitis diagnosis as comorbidities.

**Table 1:** Socio-demographic characteristics of HIV patients in Kazakhstan.

Demographic characteristics	Overall 2,213	Male	Female	*p*-value
		1,375 (62.1)	838 (37.9)	
*Age, mean ± SD*	33.3 ± 13.3	34.4 ± 12.8	31.6 ± 13.7	<0.001
*Age groups, n (%)*				<0.001
<18	426 (19.2)	247 (58.0)	179 (42.0)	
18–34	579 (26.2)	286 (49.4)	293 (50.6)	
35–44	835 (37.7)	605 (72.5)	230 (27.5)	
45–50	214 (9.7)	149 (69.6)	65 (30.4)	
>50	159 (7.2)	88 (55.4)	71 (44.6)	
*Ethnicity, n (%)*				<0.001
Kazakh	681 (30.8)	364 (53.5)	317 (46.5)	
Russian	1,092 (49.3)	719 (65.8)	373 (34.2)	
Other	399 (18.0)	268 (67.2)	131 (32.8)	
*Residence, n (%)*				0.21
Urban	1,618 (73.1)	1,018 (62.9)	600 (37.1)	
Rural	595 (26.9)	357 (60)	238 (40)	
*Hospital admission, n (%)*				<0.001
Planned	503 (22.7)	368 (73.2)	135 (26.8)	
Urgent	1710 (77.3)	1,007 (58.9)	703 (41.1)	
*All-cause mortality, n (%)*				<0.001
Alive	1,338 (60.5)	740 (55.3)	598 (44.7)	
Dead	875 (39.5)	635 (72.6)	240 (27.4)	
*Hospitalization outcome, n (%)*				<0.001
Discharged	1,455 (65.7)	827 (56.8)	628 (43.2)	
Transferred	49 (2.2)	37 (75.5)	12 (24.5)	
Refused care	59 (2.7)	41 (69.5)	18 (30.5)	
Dead	650 (29.4)	470 (72.3)	180 (27.7)	
*Treatment outcome, n (%)*				<0.001
Recovery	91 (4.1)	47 (51.6)	44 (48.4)	
Improvement	1,280 (57.8)	728 (56.9)	552 (43.1)	
Without changes	177 (8.0)	123 (69.5)	54 (30.5)	
Deterioration	15 (0.7)	7 (46.7)	8 (53.3)	
Death	650 (29.4)	470 (72.3)	180 (27.7)	
*Social status, n (%)*				0.012
Unemployed	1,214 (54.9)	762 (62.8)	452 (37.2)	
Employed	309 (14.0)	189 (61.2)	120 (38.8)	
Disabled	62 (2.8)	49 (79.1)	13 (20.9)	
Retired	25 (1.1)	10 (40)	15 (60)	
Students	375 (16.9)	219 (58.4)	156 (41.6)	
Others	228 (10.3)	146 (64)	82 (36)	
*Hospital profile*				<0.001
Non-tuberculosis (Infectious, Internal medicine, others)	1711 (77.3)	997 (58.3)	714 (41.7)	
Tuberculosis	493 (22.3)	369 (74.8)	124 (25.2)	
Comorbidities				
Viral hepatitis (B and C)	113 (5.1)	31 (27.4)	82 (72.6)	<0.001
Alcohol abuse and IDU	368 (16.6)	92 (25)	276 (75)	<0.001
Tuberculosis	479 (21.6)	159 (33.2)	320 (66.8)	<0.001

**Figure 2:**
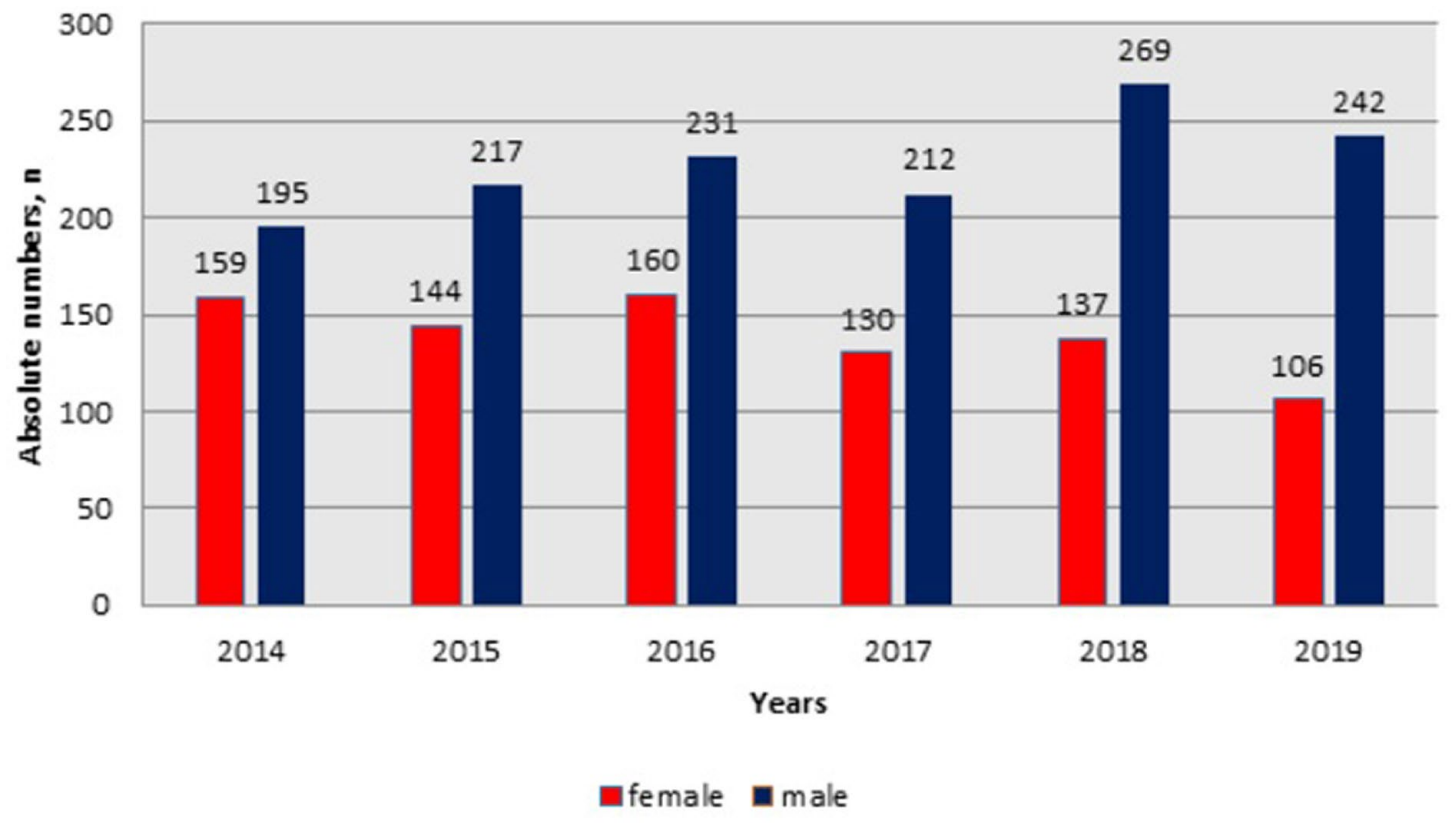
Dynamics of hospital admissions of HIV infected patients by gender in Kazakhstan between 2014-2019.

From Figure 2, the admission frequency of male HIV-infected patients increased from 195 in 2014 to 269 patients in 2018, whereas in 2019 it showed a slight decrease (n = 242). However, the admission frequency of females declined from 159 in 2014 to 106 patients in 2019.

### 3.2. Incidence, prevalence, mortality rate

**Figure 3:**
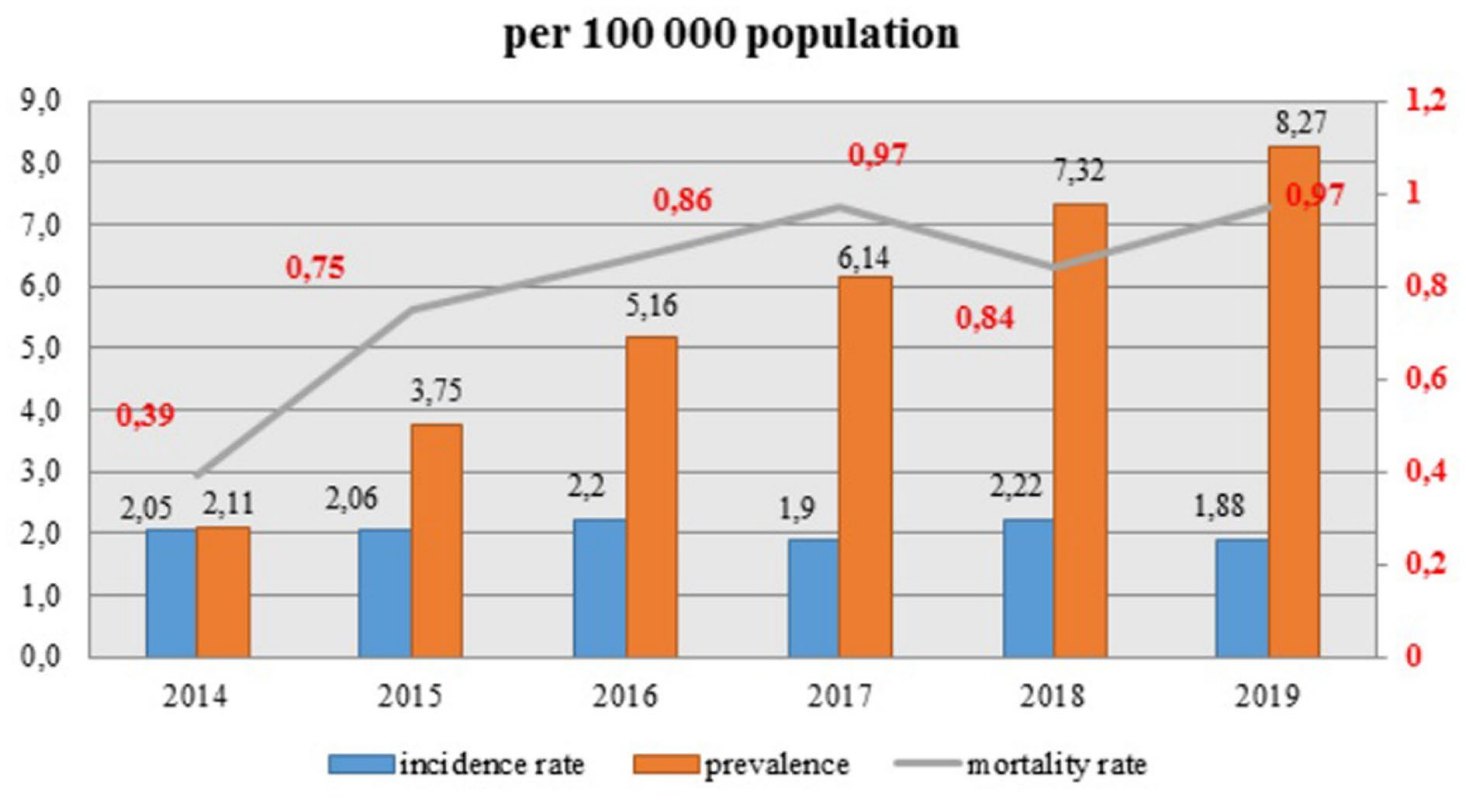
Total incidence, prevalence and mortality rate of HIV patients in 2014-2019.

Observed prevalence is increasing dramatically every year and continues to escalate, whereas the incidence rate of HIV infection remains quite stable with small fluctuations (Figure 3). Overall, incidence rate (per hundred thousand population) decreased from 2.05 in 2014 to 1.88 in 2019. However, the mortality rate raised significantly from 0.39 in 2014 to 0.97 in 2019 and it showed an increasing trend for the whole period, except for a slight decline in 2018 to 0.84.

**Figure 4:**
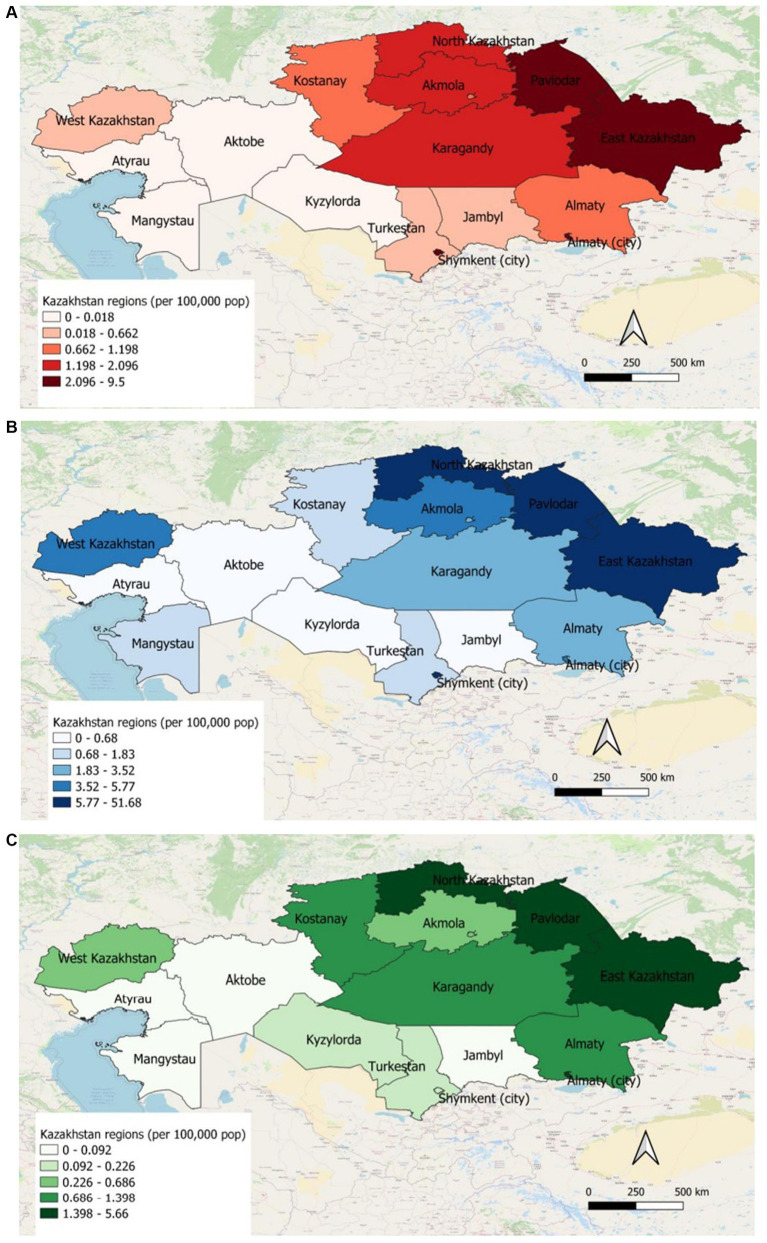
**(A)** Incidence of HIV patients in different regions of Kazakhstan for 2019. **(B)** Prevalence of HIV patients in different regions of Kazakhstan for 2019. **(C)** All-cause mortality of HIV patients in different regions of Kazakhstan for 2019.

The geographic distribution of HIV represented the highest incidence rate of hospitalized patients (2.1–9.5 per 100,000 population) and prevalence (5.77–51.68 per 100,000 population) in Pavlodar, East Kazakhstan regions and in Shymkent, Almaty cities in 2019. Whereas Atyrau, Aktobe, Mangystau and Kyzylorda regions showed the lowest incidence (0–0.018 per 100′000 population) and prevalence (0–0.68 per 100,000 population) of HIV infection in 2019. All-cause mortality of HIV-infected patients for 2019 was the highest in North Kazakhstan, Pavlodar, East Kazakhstan regions and in Almaty city (1.4–1.56 per 100,000 population) (Figures 4A–C).

### 3.3. Survival of patients with HIV

Table 2 demonstrates the crude mortality rates of HIV-infected patients per 1,000 person years (PY). People aged >50 years old showed the highest mortality rate (5330.9 per 1,000 PY) due to other comorbidities and complications. In addition, males (1331.7 per 1,000 PY) had almost 2 times higher mortality rate than females (799.3 per 1,000 PY). Mortality of HIV-infected patients of Russian ethnicity (2874.8 per 1,000 PY) was the highest among other ethnicities and people living in urban areas (1363.1 per 1,000 PY) showed much higher mortality than people living in rural areas (691.1 per 1,000 PY). In regards to social status, retired, disabled and unemployed people represented a high proportion of mortality rate. It is observed that people admitted to tuberculosis hospitals (6189.2 per 1,000 PY) with tuberculosis as comorbidity (3347.8 per 1,000 PY) had much higher mortality rate. As for the geographical distribution, West and Central Kazakhstan regions demonstrated the highest mortality rates, 13672.5 and 12759.8 per 1,000 PY, respectively.

**Table 2:** Crude mortality rates of HIV-infected patients.

	Overall 2,213	Alive	Dead	*p*-value	Mortality rate per 1,000 PY [95% CI]
		1,338 (60.5)	875 (39.5)		
Age, mean ± SD	33.3 ± 13.3	29.5 ± 14.1	39.1 ± 9.1	<0.001	
Age groups, n (%)					
<18	426 (19.2)	413 (96.9)	13 (3.1)	<0.001	28.0 [14.6; 53.8]
18–34	579 (26.2)	340 (58.7)	239 (41.3)	<0.001	3255.7 [2796.3; 3790.7]
35–44	835 (37.7)	420 (50.3)	415 (49.7)	<0.001	2611.6 [2315.9; 2945.1]
45–50	214 (9.7)	100 (46.7)	114 (53.3)	<0.001	2131.2 [1699.5; 2672.4]
>50	159 (7.2)	65 (40.9)	94 (59.1)	<0.001	5330.9 [4156.2; 6837.5]
Gender					
Female	838 (37.9)	598 (71.4)	240 (28.6)	<0.001	799.3 [688.9; 927.4]
Male	1,375 (62.1)	740 (53.8)	635 (46.2)	<0.001	1331.7 [1207.9; 1468.1]
Ethnicity					
Kazakh	681 (30.8)	510 (74.9)	171 (25.1)	<0.001	447.7 [372.6; 537.9]
Russian	1,092 (49.3)	558 (51.1)	534 (48.9)	<0.001	2874.8 [2595.9; 3183.6]
Other	399 (18.0)	236 (59.1)	163 (40.9)	<0.001	1324.7 [1085.7; 1616.4]
Residence					
Urban	1,618 (73.1)	915 (56.6)	703 (43.4)	<0.001	1363.1 [1241.7; 1496.2]
Rural	595 (26.9)	423 (71.1)	172 (28.9)	<0.001	691.1 [584.2; 817.6]
Social status					
Unemployed	1,214 (54.9)	585 (48.2)	629 (51.8)	<0.001	3125.0 [2830.1; 3450.6]
Employed	309 (14)	208 (67.3)	101 (32.7)	<0.001	1664.9 [1319.4; 2100.9]
Disabled	62 (2.8)	26 (41.9)	36 (58.1)	0.338	3267.5 [2171.3; 4917.0]
Retired	25 (1.1)	9 (36)	16 (64)	0.155	4054.9 [2354.5; 6983.3]
Students	375 (16.9)	368 (98.1)	7 (1.9)	<0.001	16.8 [6.9; 40.3]
Others	228 (10.3)	142 (62.3)	86 (37.7)	<0.001	1671.2 [1332.7; 2095.6]
Hospital profile					
Non-tuberculosis	1711 (77.3)	1,230 (71.9)	481 (28.1)	<0.001	813.6 [733.8; 902.2]
Tuberculosis	493 (22.3)	103 (20.9)	390 (79.1)	<0.001	6189.2 [5418.2; 7070.0]
Region					
Central KZ	114 (5.2)	26 (22.8)	88 (77.2)	<0.001	12759.8 [9023.4; 18043.4]
South KZ	929 (42)	631 (67.9)	298 (32.1)	<0.001	478.2 [409.7; 558.0]
North KZ	334 (15.1)	141 (42.2)	193 (57.8)	<0.001	5202.5 [4421.0; 6122.1]
West KZ	56 (2.5)	38 (67.9)	18 (32.1)	<0.001	13672.5 [8097.5; 23085.5]
East KZ	778 (35.2)	499 (64.1)	279 (35.9)	<0.001	2082.8 [1829.8; 2370.8]
Comorbidities					
Viral hepatitis	113 (5.1)	85 (75.2)	28 (24.8)	<0.001	1155.5 [774.5; 1724]
Alcohol abuse and intravenous drug use	368 (16.6)	182 (49.5)	186 (50.5)	<0.001	2176.2 [1816.9; 2606.5]
Tuberculosis	479 (21.6)	119 (24.8)	360 (75.2)	<0.001	3347.8 [2910.4; 3850.9]

**Figure 5:**
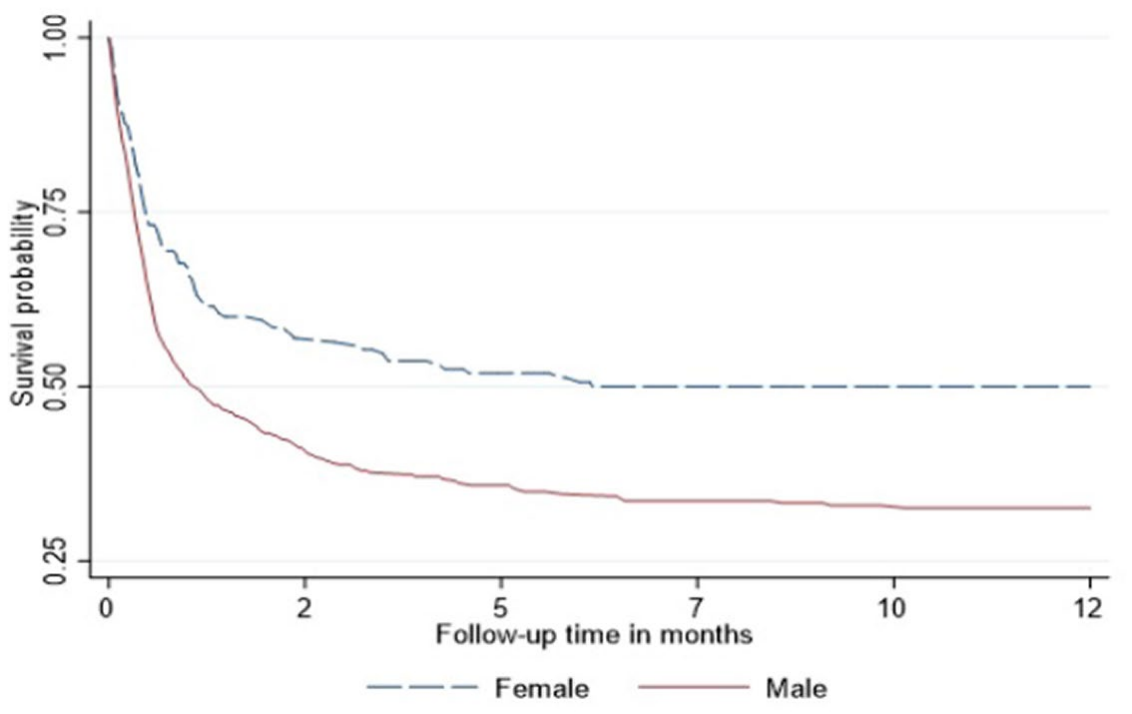
Kaplan-Meier survival function among HIV patients adjusted for gender.

**Figure 6:**
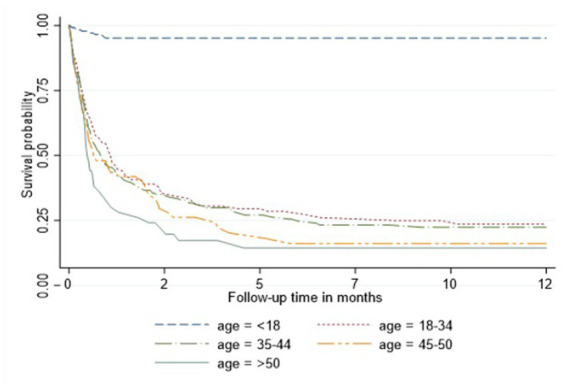
Kaplan-Meier survival function among HIV patients adjusted for age.

**Figure 7:**
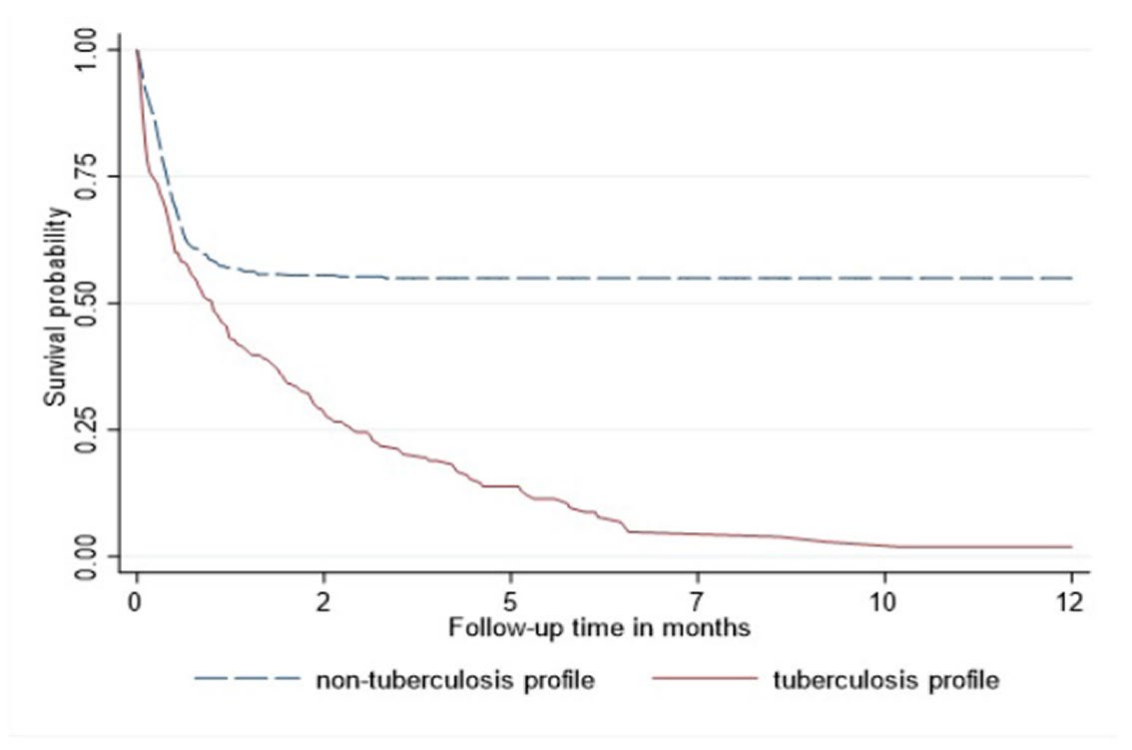
Kaplan-Meier survival function among HIV patients adjusted for hospital profile.

In the Cox proportional hazard model (Table 3), people >50 years old had 9.6, the highest hazard of death than other age groups in the adjusted model. Retired people, correspondingly, had a higher hazard ratio (14.4) than other social status categories (Table 3). Males had 60% higher risk of death than females both in unadjusted and adjusted models. According to geographical distribution, Central Kazakhstan demonstrated the highest hazard of death (1.6) among other regions of Kazakhstan in a multivariable model. Patients with tuberculosis represented 40% higher risk of death in adjusted PH model. Whereas people coinfected with viral hepatitis and alcohol abuse along with IDU showed non-significant results (Table 3). The Kaplan–Meier survival functions (Figures 5–7) also estimated that females have better survival probability than males, moreover, survival probability declines as people get older. According to hospital profiles, HIV-infected patients from the tuberculosis hospital profile had much worse survival than non-tuberculosis profiles.

**Table 3:** Results of multivariable Cox regression analysis of HIV patients in Kazakhstan.

Variables	Unadjusted HR [95% CI]	*p*-value	Adjusted HR^*^ [95% CI]	*p*-value
Age groups				
<18	ref		ref	
18–34	24.3 [12.4; 47.7]	<0.001	8.6 [3.4; 21.6]	<0.001
35–44	26.3 [13.5; 51.2]	<0.001	7.9 [3.2; 19.7]	<0.001
45–50	30.4 [15.2; 60.7]	<0.001	9.1 [3.6; 23.1]	<0.001
>50	35.3 [17.5; 71.3]	<0.001	9.6 [3.7; 24.7]	<0.001
Gender				
Female	ref		ref	
Male	1.6 [1.3; 1.9]	<0.001	1.6 [1.4; 2.0]	<0.001
Regions				
South KZ	ref		ref	
East KZ	1.8 [1.5; 2.2]	<0.001	0.6 [0.5; 0.7]	<0.001
North KZ	2.5 [2.0; 3.1]	<0.001	0.7 [0.6; 1.0]	0.027
West KZ	2.7 [1.5; 4.6]	<0.001	1.2 [0.7; 2.1]	0.492
Central KZ	4.8 [3.2; 7.0]	<0.001	1.6 [1.0; 2.3]	0.032
Hospital profile				
Non-tuberculosis	ref		ref	
Tuberculosis	2.2 [1.9; 2.6]	<0.001	1.1 [0.9; 1.4]	0.4
Social status				
Students	ref		ref	
Employed	30.5 [12.2; 75.8]	<0.001	5.7 [1.7; 18.9]	0.005
Others	34.7 [14.0; 85.8]	<0.001	8.2 [2.6; 26.5]	<0.001
Disabled people	41.9 [15.9; 110.9]	<0.001	7.1 [2.0; 24.8]	0.002
Unemployed	44.9 [18.5; 108.7]	<0.001	7.7 [2.3; 25.2]	<0.001
Retired people	71.9 [25.6; 202.1]	<0.001	14.4 [3.8; 55.0]	<0.001
Comorbidities				
Viral hepatitis	0.8 [0.5; 1.2]	0.276	0.7 [0.4; 1.0]	0.057
Alcohol abuse and intravenous drug use	1.4 [1.1; 1.7]	0.002	1.1 [0.9; 1.3]	0.497
Tuberculosis	2.3 [1.9; 2.7]	<0.001	1.4 [1.1; 1.7]	<0.001

^*^Adjusted for demographic, clinical and behavioral characteristics along with comorbidities.

## 4. Discussion

This study provides a comprehensive assessment of dynamics of hospital admissions of HIV-infected patients in Kazakhstan. Analysis indicated that prevalence and mortality of HIV continually increased each year in Kazakhstan, and showed regional, age, gender, hospital profile and social status significant differences. Thus, it requires controlling the spread of HIV infection in Kazakhstan which needs implementation of more effective prevention strategies to reduce HIV incidence.

Results demonstrate that middle aged people (35–44 years) are the most important risk groups in terms of transmitting HIV infection in Kazakhstan. The age distribution varies considerably in different studies, but in most studies sexually active young people aged 20–45 years are in risk groups. The HIV infection is diagnosed at younger ages mostly in countries where the incidence of HIV is high and in many developed countries (8). In our study the mean age of patients was 33.3 ± 13.3 years. However, mortality of patients aged >50 living with HIV is relatively high. This is the result of low availability of effective ART and other challenges that older people face, such as chronic diseases, cognitive and mental comorbidities, physical frailty and deconditioning (9).

Males comprise the majority of HIV patients in published studies from US, Turkey, China and Sub-Saharan Africa (8–12). Our results indicate that almost two-thirds of the patients (62%) were males which coincide with the global trend. HIV infection is more prevalent among males due to engagement in high-risk behavior leading to transmission (8).

All-cause mortality rates for the cohort was relatively high (almost 40%) and most patients hospitalized urgently, it suggests that the disease is diagnosed in more advanced periods or in the terminal stages. It can be the result of low coverage of population testing for HIV infection in Kazakhstan. According to the report on prevention of infectious diseases and treatment of HIV/AIDS and hepatitis among injecting drug users in Central Asia, in 2017 the coverage of population testing was only 13.9% in Kazakhstan (13). This is because patients did not attend check-ups due to the social, mental factors and stigma associated with HIV.

There has been an increase in the prevalence of HIV in the period of 2014–2019, the highest prevalence observed in 2019 (8.27). Correspondingly, there has been an increase in the mortality rate during this period of follow-up from 0.39 in 2014 to 0.97 in 2019. The main cause of the mortality in HIV-infected patients is late presentation to health institutions in most countries around the world (8). Since HIV/AIDS is still a disease with no curative treatment, the mortality rate remains high in most countries. In our study, there was a diversity in geographical distribution. Geographical maps show high prevalence and mortality in North, East Kazakhstan and Pavlodar regions, also in Almaty and Shymkent cities. The outbreak of massive HIV infection in Shymkent city in 2006 demonstrated hierarchic negligence of the health care system of Kazakhstan. 149 children were infected by blood transfusion as a result of medical staff’s dereliction of duty. Even though the prevalence of HIV in Shymkent remains to be high, the mortality rate is relatively low, due to close monitoring of outbreak HIV patients and treatment with ART (10, 14). However, the substandard health care system and low monitoring of infection in North and in East regions of Kazakhstan mandates urgent attention and investigation of causes, routes of transmission and other characteristics, in order to halt the spread of HIV epidemic by creating important preventive policies. This is not an easy task for a post-soviet healthcare system such as Kazakhstan, with an uneven distribution of healthcare expenditure between the maternal and child care, tuberculosis dyspancers, overhospitalization, and little attention given to prevention and vast screening of HIV. On the upside, the major part of medical care is provided during hospitalizations for urgent, and planned admissions for management of chronic diseases or wellness checkups. One of the requirements of hospital admission is HIV screening, which gives an opportunity to gather valid information on HIV-infected patients population and hospitalization dynamics in the country.

The most commonly reported opportunistic HIV co-infections were candidiasis, PCP, and tuberculosis (15–18). The incidence of tuberculosis was greater in other studies, as well as in our study. Tuberculosis is endemic and is considered as a major public health problem in Kazakhstan. According to WHO, the incidence rate of TB was 67 new and relapse cases per 100,000 population in 2016 (19, 20). In order to reduce the HIV mortality, it is necessary to prevent, early detect and to treat opportunistic infections. Coinfection of HIV and tuberculosis is the leading cause of death among people living with HIV. That is why, it is crucial to manage the strategy to increase the linkage and co-treatment between HIV and tuberculosis. According to the report on prevention of infectious diseases and treatment of HIV/AIDS and hepatitis among injecting drug users in Central Asia, Kazakhstan is one of 15 countries with the highest burden of multidrug resistant tuberculosis (MDR-TB) in the European region (13). Thus, in all TB detected cases, counseling and screening for HIV infection needs to be provided.

In view of the fact that the primary route of transmission of HIV infection in Kazakhstan is IDU, it is important to consider the risk of coinfection with other blood-borne pathogens such as Hepatitis C and B viruses (21, 22). However, the results of our studies represented a non-significant concomitance with viral hepatitis, but all-cause mortality of HIV patients among alcohol abuse and IDU patients was higher. Therefore, strategies to prevent transmission of the HIV infection by injecting drugs and substance abuse might have broader public health impact in terms of prevention of blood-borne pathogens.

This study faced several limitations. As this data obtained from the hospital admissions, it does not cover the whole HIV population in Kazakhstan, thus, the prevalence of HIV infected patients in our cohort cannot represent the real number of patients over the country. Low rate of HIV testing due to fear of stigma associated with the disease puts our society at a high risk of HIV infection and prevents infected patients from initiating early HIV treatment. Moreover, the deficiency of epidemiological data on HIV may result in bias in this study. The data is incomplete on route of transmission, other risk factors, drug use, prostitution and blood donation which is important for diagnosing HIV and implementing appropriate care. Results of multivariate analysis depend on the availability of collected information. Although the completion rates were relatively high for the used database with very low missed data (<5%), it was difficult to extract data on comorbidities. During linkage of different databases for coinfections some patients may have been missed. Therefore, the impact of comorbidities in the survival multivariable model may vary. Despite these limitations, this is the first study that analyses hospital admissions of HIV-infected patients and focuses on regional, gender, age, hospital profile and social status differences, as well as with other comorbidities. It is strongly recommended to develop behavioral surveillance data on known risk behaviors (eg. sex work and drug use), as well as surrogate markers of HIV risk, such as sexually transmitted infections. This is necessary so that intervention programs can be properly selected and evaluated.

## 5. Conclusion

This study was performed to assess the dynamics of hospital admissions of HIV infected patients in Kazakhstan. The results of this study demonstrate high rates of HIV mortality and strong association of HIV with TB coinfections. It also concludes that regional, age specific, gender, hospital profile and social status differences of HIV prevalence significantly existed. Since the prevalence of HIV is continuing to increase, more information is necessary for evaluation and implementation of prevention procedures. It is considered that most patients were diagnosed at late stages of the disease due to the stigma, which confirms the importance of early diagnosis. Thus, more efficient and effective control programs of HIV transmission need to be investigated.

## Data availability statement

The data analyzed in this study is subject to the following licenses/restrictions: not publicly available. Requests to access these datasets should be directed to Republican Center for Electronic Health of the Ministry of Health of the Republic of Kazakhstan.

## Author contributions

KM, AnA, YS, DS, ArG, AinA, AiyA, and AbG were involved in creating the study protocol. KM, AnA, and AbG drafted the manuscript. KM, AnA, DS, ArG, and YS involved in preliminary data design and assessment, literature review. ArG and YS collected data. AinA, AiyA, and AbG provide a critical revision and regular feedback of the manuscript. KM, AnA, YS, DS, ArG, AinA, AiyA, and AbG contributed to refinement of the study protocol. All authors contributed to the article and approved the submitted version.

## Funding

This study was supported by grants from the Ministry of Science and Higher Education of the Republic of Kazakhstan 2021–2023 (Funder Project Reference: AP09259016). The funder had no role in study design, data collection and analysis, decision to publish, or preparation of the manuscript. AbG is a PI of the projects.

## Conflict of interest

The authors declare that the research was conducted in the absence of any commercial or financial relationships that could be construed as a potential conflict of interest.

## Publisher’s note

All claims expressed in this article are solely those of the authors and do not necessarily represent those of their affiliated organizations, or those of the publisher, the editors and the reviewers. Any product that may be evaluated in this article, or claim that may be made by its manufacturer, is not guaranteed or endorsed by the publisher.
